# Adaptation of Respiratory-Related Brain Regions to Long-Term Hypercapnia: Focus on Neuropeptides in the RTN

**DOI:** 10.3389/fnins.2019.01343

**Published:** 2019-12-13

**Authors:** Ayse Sumeyra Dereli, Zarwa Yaseen, Pascal Carrive, Natasha N. Kumar

**Affiliations:** ^1^Department of Pharmacology, School of Medical Sciences, University of New South Wales, Sydney, NSW, Australia; ^2^Department of Anatomy, School of Medical Sciences, University of New South Wales, Sydney, NSW, Australia

**Keywords:** chemoreception, neuropeptide, hypercapnia, neuroplasticity, respiratory, retrotrapezoid nuclus, c-Fos

## Abstract

Long-term hypercapnia is associated with respiratory conditions including obstructive sleep apnea, chronic obstructive pulmonary disease and obesity hypoventilation syndrome. Animal studies have demonstrated an initial (within hours) increase in ventilatory drive followed by a decrease in this response over the long-term (days–weeks) in response hypercapnia. Little is known about whether changes in the central respiratory chemoreflex are involved. Here we investigated whether central respiratory chemoreceptor neurons of the retrotrapezoid nucleus (RTN), which project to the respiratory pattern generator within the ventral respiratory column (VRC) have a role in the mechanism of neuroplasticity associated with long-term hypercapnia. Adult male C57BL/6 mice (*n* = 5/group) were used. Our aims were (1) to determine if galanin, neuromedin B and gastrin-releasing peptide gene expression is altered in the RTN after long-term hypercapnia. This was achieved using qPCR to measure mRNA expression changes of neuropeptides in the RTN after short-term hypercapnia (6 or 8 h, 5 or 8% CO_2_) or long-term hypercapnia exposure (10 day, 5 or 8% CO_2_), (2) in the mouse brainstem, to determine the distribution of preprogalanin in chemoreceptors, and the co-occurrence of the galanin receptor 1 (GalR1:Gi-coupled receptor) with inhibitory GlyT2 ventral respiratory column neurons using *in situ* hybridization (ISH) to better characterize galaninergic RTN-VRC circuitry, (3) to investigate whether long-term hypercapnia causes changes to recruitment (detected by cFos immunohistochemistry) of respiratory related neural populations including the RTN neurons and their galaninergic subset, *in vivo*. Collectively, we found that hypercapnia decreases neuropeptide expression in the RTN in the short-term and has the opposite effect over the long-term. Following long term hypercapnia, the number of RTN galanin neurons remains unchanged, and their responsiveness to acute chemoreflex is sustained; in contrast, we identified multiple respiratory related sites that exhibit blunted chemoreflex activation. GalR1 was distributed in 11% of preBötC and 30% of BötC glycinergic neurons. Our working hypothesis is that during long-term hypercapnia, galanin co-release from RTN neurons may counterbalance glutamatergic inputs to respiratory centers to downscale energetically wasteful hyperventilation, thereby having a role in neuroplasticity by contributing to a decrease in ventilation, through the inhibitory effects of galanin.

## Introduction

Respiratory conditions including chronic obstructive pulmonary disease (COPD), obesity hypoventilation syndrome (OHS) and obstructive sleep apnea (OSA) are associated with long-term hypercapnia and hypoxia. Associated long-term detrimental effects include poor quality of life, poor cognitive function and increased mortality rate. While the changes in the respiratory chemoreflex mechanisms during long-term hypoxia are extensively investigated (Peng et al., 2001, 2003, 2006; Rey et al., 2004; Huang et al., 2009; Morgan et al., 2016; Barnett et al., 2017) little is known about long-term hypercapnia. Acute hypercapnia causes an increase in ventilatory drive by peripherally and centrally mediated chemoreflex mechanisms (Forster and Smith, 2010; Smith et al., 2010). Long-term hypercapnia shows a biphasic ventilatory response in humans, dogs, goats and rodents, consisting of an initial increase in ventilatory drive in the first 8 h (rodents), 24 h (goats and dogs), 5 days (human) followed by a sustained decrease in this response (21–44 days) (Schaefer, 1963; Schaefer et al., 1963; Clark et al., 1971; Pingree, 1977; Lai et al., 1981; Jennings and Davidson, 1984; Burgraff et al., 2018; Burgraff et al., 2019). The mechanism underlying physiological adaptation to long-term hypercapnia is not clearly understood. Many peripheral factors are suggested to contribute to this plasticity including metabolic compensation, muscle fiber transformation in the diaphragm and changes in lung hyaline membrane turnover (Schaefer et al., 1964; Lai et al., 1981; Kondo et al., 2000; Johnson, 2017; Burgraff et al., 2018). Lai et al. (1981) first suggested a contribution from central chemoreceptors to this adaptation; more recently, changes in glutamate receptor expression were observed in central chemoreceptors (Burgraff et al., 2019).

The retrotrapezoid nucleus (RTN) chemoreceptor neurons are intrinsically sensitive to brain pH/Pco_2_ (Takakura et al., 2006; Wang S. et al., 2013) and are critical in mediating the central respiratory chemoreflex. RTN neurons send excitatory drive to the ventral respiratory column (VRC) via extensive projections (Mulkey et al., 2004; Abbott et al., 2009b; Bochorishvili et al., 2012). The VRC generates the rhythmic breathing pattern and regulates the depth and frequency of breathing through innervation of motor neurons that control the muscles of breathing (Feldman et al., 2003; Nattie and Li, 2012).

It is clear that glutamatergic neurotransmission from the RTN confers CO_2_ stimulated breathing (Holloway et al., 2015), however, RTN neurons also express a distinctive peptidergic phenotype. All RTN neurons contain mRNA for neuromedin B (NMB) and pituitary adenylate-activating polypeptide (PACAP) (Shi et al., 2017), 50–70% express galanin (Stornetta et al., 2009; Shi et al., 2017), and a subset express gastrin-releasing peptide (GRP) (Li et al., 2016). Furthermore, the RTN has distinct functional subpopulations of neurons; for example, pre-inspiratory oscillatory neurons provide rhythmic excitatory drive (Onimaru et al., 1997; Smith et al., 2009), neurons lateral to the facial nucleus are involved in active expiration (Pagliardini et al., 2011) and ventral to parafacial neurons are chemosensitive (Huckstepp et al., 2015). The expression of specific neuropeptides by the RTN neurons might further elucidate the mechanism of these functionally distinct RTN subpopulations.

Various functional studies have indicated the role of inducible neuropeptides in the control of breathing. While NMB and GRP are excitatory (Moody and Merali, 2004; Dickson et al., 2006; Roesler and Schwartsmann, 2012), galanin is an inhibitory neuropeptide (Lang et al., 2015). Microinjection of galanin into the VRC induces apnea by inhibiting phrenic nerve activity and ventilatory chemoreflex responses (Abbott et al., 2009a) and injection of NMB and GRP into the VRC causes sighing (Li et al., 2016). Neuropeptides are known to have long-lasting effects since they act on G-protein coupled receptors (Salio et al., 2006; Nassel, 2009). Therefore, long term adjustments in neuropeptide co-release are likely important in fine-tuning the fast transmitter output of respiratory neuronal circuits in response to long-term changes in blood gas levels. Such long-term changes may occur during chronic respiratory disorders (such as hypercapnia and hypoxia related COPD, OHS, OSA, etc.) or following long-term exposure to altered environmental gas conditions (such as hypoxia at high altitudes, or hypercapnia in submarines, caves, space shuttles or mines).

The impact of long-term hypercapnia on the central respiratory chemoreflex and the contribution of neuropeptides has not been explored. Following chronic exposure (35 days) to elevated inspired CO_2_ (1.5% CO_2_), healthy human subjects show a significant depression of the respiratory response to 15 min inhalation of 5% CO_2_ (Schaefer, 1963; Schaefer et al., 1963). Furthermore, there is a decreased ventilatory response of respiratory disease patients to acute inspired hypercapnia, suggesting a decline in their central CO_2_/H^+^ chemoreflex system (Kepron and Cherniack, 1973; Montes De Oca and Celli, 1998). The underlying mechanism for this blunted ventilatory response to acute hypercapnia following exposure to long-term hypercapnia is not clear. Altered chemosensory responsiveness, or adjusted peptidergic transmission by RTN neurons may explain this change in respiratory behavior following long-term hypercapnia.

In light of these gaps in knowledge, we hypothesize that neuropeptides have a role in adaptation of central respiratory chemoreception and may be involved in the pathophysiology of respiratory disorders associated with long-term hypercapnia. Our aims are (1) to determine if neuropeptide gene expression is altered in the RTN after long-term hypercapnia. This is achieved using quantitative polymerase chain reaction (qPCR) to determine mRNA expression changes in the RTN after short-term hypercapnia (6 or 8 h exposure to 5 or 8% CO_2_) or long-term hypercapnia (10 days exposure to 5 or 8% CO_2_), (2) to determine the distribution of preprogalanin in chemoreceptors in the mouse brainstem by *in situ* hybridization (ISH), and the co-occurrence of the galanin receptor 1 (GalR1:Gi-coupled receptor) in VRC neurons using multiplex fluorescent ISH (FISH) (RNAscope, ACD BioScience, Hayward, CA, United States) to better characterize galaninergic VRC circuitry, (3) to investigate whether long-term hypercapnia causes changes to recruitment of known respiratory related neuronal populations (detected by c-Fos immunohistochemistry) including RTN neurons and the galaninergic subset, *in vivo*.

Collectively, we found that hypercapnia (short-term and long-term) alters neuropeptide expression in the RTN; we decided to follow up the observed changes in preprogalanin expression. GalR1 was distributed throughout the VRC, including in 9% of glycinergic VRC neurons. We identified multiple respiratory-related populations that exhibit blunted chemoreflex activation following long-term hypercapnia. However, RTN neurons (pre-eminently galaninergic subset) retained their responsiveness to the chemoreflex following long-term hypercapnia.

## Materials and Methods

### Animals

All experiments in this project were conducted in accordance with the National Health and Medical Research Council (Australia) with the approval by the University of New South Wales Animal Care and Ethics Committee. The animals used were male adult C57BL/6J mice (25–30 g). All animal experiments were conducted in the Biological Resources Centre (University of New South Wales, Sydney, Australia). They were maintained on 12 h:12 h light:dark cycle at 23°C with standard chow and tap water available *ad libitum* and housed in conventional caging.

### Respiratory Paradigms

#### Short-Term and Long-Term Hypercapnia Paradigms

**FIGURE 1:**
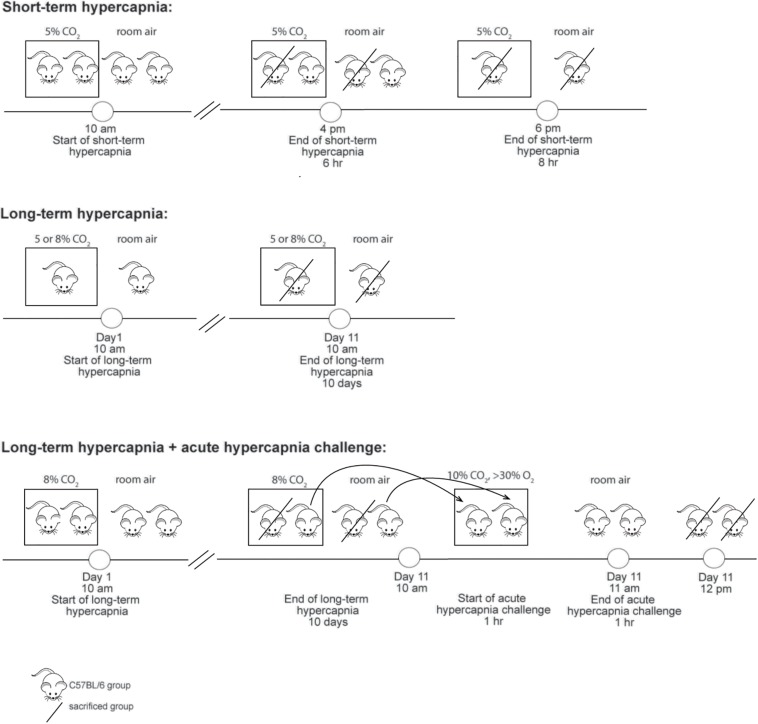
Timeline summarizing different experimental paradigms.

Mice were randomly assigned to either short-term hypercapnia (SH), long-term hypercapnia (LH) or room air (RA) (*n* = 5/group). Hypercapnia was achieved by placing the animals within their home cages in a sealed chamber measuring 9000 cm^3^ (Biospherix, NY, United States). A CO_2_ monitor was connected, that continuously flushed a designated amount of CO_2_ into the chamber. Animals were acclimatized to the experimental room or the hypercapnia chamber for 1 h a day over 2 days in order to minimize stress and non-specific gene expression related to novel environment. Then, the CO_2_ monitor was set to either 5 or 8% CO_2_ (balanced with room air, 20 ± 0.5% O_2_) at 15 psi. See Figure 1 for the timeline. During all exposures, O_2_ and CO_2_ concentrations were continuously monitored by capnometry (Normocap oxy, Datex Engstrom, WI, United States) and CO_2_ meter (GM70, Vaisala, Finland). Room air mice were left outside the chamber in the experimental room for the same period of time. At the end of the paradigm, animals were deeply anaesthetized with sodium pentobarbital (70 mg/kg, ip) followed by immediate isolation of the brain (qPCR) or transcardial perfusion with heparinised 0.1M phosphate buffered saline (PBS) and 4% paraformaldehyde (PFA, Sigma-Aldrich, NSW) in 0.1M sodium phosphate buffer (ISH/IHC).

#### c-Fos Studies: Acute Hypercapnic Chemoreflex Challenge (AH)

A subset of long-term hypercapnia or room air animals were exposed to 10% CO_2_, 30–40% O_2_ balanced with N_2_ for 1 h then left in room air for 1 h (Figure 1) before being deeply anaesthetized followed by transcardial perfusion as described above. Hyperoxic conditions were used for the acute hypercapnic chemoreflex challenge because this silences the peripheral chemoreceptors (Lahiri et al., 1987), ensuring that the ventilatory response is mediated by central mechanisms.

### Quantitative PCR

Following isolation of the brainstem, a cut was made separating the dorsal brainstem from the ventral brainstem. The rostral parafacial region containing rostral RTN (rRTN: 0.7 mm rostral, 0.5 mm caudal to the trapezoid line, Bregma −5.3 to −6.5, midline omitted), caudal parafacial region containing caudal RTN (cRTN: 1 mm immediately caudal to the rRTN, Bregma −6.5 to −7.5, midline omitted), left lateral cerebellum were excised, rapidly frozen in liquid nitrogen and stored at −80°C. Following RNA isolation (Promega SV Total RNA isolation kit, WI, United States), a cDNA library was generated by subjecting RNA (1000 ng) to SuperScriptTM III First Strand Synthesis System (Invitrogen, MA, United States), according to the manufacturer’s instructions. qPCR was carried out using KAPA SYBR FAST kit (Kapa Biosystems, MA, United States) in a total reaction volume of 20 μl containing 1 μl cDNA, 10 μl of qPCR mastermix and 1 μl (10 nM) forward and reverse primer. Primer sequences for neuropeptide mRNA (ppGal, ppNMB and ppGRP) (Table 1) were designed using Primer3 (v.0.4.0, Whitehead Institute for Biomedical Research). qPCR reaction was performed with Eppendorf Mastercycler^®^ ep realplex using initial denaturation (3 min 95°C), followed by 40 cycles of denaturation (95°C for 10 s), annealing (60–64°C for 20 s), and extension (72^*o*^C for 20 s). PCR products were verified by melt curve analysis and separation on a 2% agarose gel. We performed qPCR on samples in the absence of reverse transcriptase and non-template controls concurrently, to control for genomic DNA contamination. Sequencing analysis (Garvan Molecular Genetics, Sydney) confirmed that the DNA fragments generated by PCR were identical to the mRNA sequences reported in the GenBank database (Table 1). The cycle threshold (Ct) values were used to calculate mRNA expression of ppGal, ppNMB, and ppGRP relative to the expression of two house-keeping genes (HKG), hypoxanthine phosphoribosyltransferase (HPRT), and ribosomal protein lateral stalk subunit P0 (RPLPO) using the ΔΔCt method (Pfaffl, 2001). HKG mRNA was expressed at a similar abundance to the genes of interest and standard curve analysis was initially performed to assure their stability in the brain following hypercapnia used in this study. Lateral cerebellum does not have a function in the control of respiration (Xu and Frazier, 2000); therefore, it was used as a control tissue. Furthermore, to decrease bias in the experimental results, the person quantifying experimental outcomes was blinded to the experimental groups.

**TABLE 1:** Details of primers used for qPCR and for generating non-radioactive ISH riboprobes.

	**Species**	**Primer**	**Genebank ref no**	**Sequence (5′→3′)**	**Amplicon length (bp)**	**Annealing temp (^*o*^C)**	**Extension time (sec)**
For qPCR	mouse	Galanin	NM_010253.4	F: CATGCCATTGACAACCACAG	331	61	20
				R: CGATTGGCTTGAGGAGTTGG			
	mouse	NMB	NM_026523.4	F: GGCGACCGGTCACTTCAT	191	61	20
				R: GCCTCCTGTACTGGATTGG			
	mouse	GRP	NM_175012.4	F: CACGGTCCTGGCTAAGATGTAT	418	61	20
				R: CCAGTAGAGTTGACGTTTGCAGA			
	mouse	HPRT	NM_013556.2	F: TCTTTGCTGACCTGCTGGATTACAT	228	61	20
				R: CCAGGGAAAGCAAAGTTTGCATT			
	mouse	RPLPO	NM_007475.5	F: CCCTGCACTCTCGCTTTCTGGA	212	61	20
				R: AGGGGCAGCAGCCGCAAATG			
For ISH riboprobes	mouse	Galanin	NM_010253.4	FggatccatttaggtgacactatagaagCACCGAGAGAGCCTTGATCCTG^∗^	526	60	45
				R:gaattctaatacgactcactatagggagaACGATTGGCTTGAGGAGTTGG^∗∗^			
	mouse	GalR1	NM_008082.2	F:ggatccatttaggtgacactatagaagATGTCTGTGGATCGCTACGT^∗^	568	64	45
				R:gaattctaatacgactcactatagggagaTGAACACTTGCTTGTACGCC^∗∗^			

**^∗^ Lowercase base sequences indicate the SP6 universal promoter. *^∗∗^ Lowercase base sequences indicate T7 universal promoter.***

### Non-radioactive Chromogenic *in situ* Hybridization Combined With Immunohistochemistry

Fixed brains were vibratome sectioned coronally (30 μm) (Leica VT1200S, Leica, Germany) and stored in cryoprotectant solution (30% RNase free sucrose, 30% ethylene glycol, 1% polyvinylpyrrolidone in 0.1M sodium phosphate buffer, pH 7.4) at −20°C.

A template cDNA library was generated by reverse transcription of total RNA isolated from mouse brain (Kumar et al., 2012). ISH riboprobes were synthesized from cDNA which was amplified by scaled-up PCR reaction. The list of PCR primers used (with either T7 or SP6 RNA polymerase promoter regions attached) can be found in Table 1. Following purification by gel extraction of the amplified cDNA templates, antisense and sense (control) riboprobes were prepared by *in vitro* transcription with digoxigenin-11-UTP (Roche Applied Science, Mannheim, Germany) using the AmpliScribe T7 FLASH transcription kit (Epicentre Biotechnologies, Madison, WI, United States) and SP6 RiboMAX large-scale RNA production system (Promega, Madison, WI, United States), respectively.

For digoxigenin (DIG) labeled ISH assay, free floating brainstem sections were assayed as described previously (Kumar et al., 2012; Spirovski et al., 2012). Briefly, riboprobes were added to the hybridization solution (500–1000 ng/μl) for an overnight incubation at 58°C. The sections were then rinsed through a series of decreasing salt concentrations and blocked prior to primary antibody incubation (24–48 h at 4°C). The antibodies and concentrations used are summarized in Table 2 and include sheep-anti DIG (1:1000, tagged with alkaline phosphatase, Roche). Sections were then rinsed with tris buffered saline (TBS) buffer and secondary antibodies were added at 1:400, diluted in TBS buffer containing 1% NHS and 0.1% Tween-20, and incubated at 4°C overnight. Following detection of cytoplasmic DIG labeling, sections were mounted and coverslipped with mounting medium with DAPI (Fluoroshield, Sigma, Australia) or without DAPI (Fluoromount Aqueous Mounting Medium, Sigma, Australia).

**TABLE 2:** Antibodies used for IHC.

**Antibody**	**Host**	**Working dilution**	**Company (Cat no)**	**Specificity and related PMID**	**Associated figures**
**PRIMARY**					
DIG	Sheep	1:1000	Roche (11093274910)		
Phox2b	Guinea pig	1:1500	Gift from Professor Hideki Enomoto (Nagashimada et al., 2012)	Confirmed by co-expression with rabbit anti-Phox2b (J.-F. Brunet, Ecole Normale Supeìrieure, Paris, France), for which there was complete absence of reactivity in Phox2b knockout mice (Lazarenko et al., 2009; Nagashimada et al., 2012)	**F 6A–D**
c-Fos	Rabbit	1:1000	Cell Signaling (2250S)	29888787	**F 6A–D**
c-Fos	Rabbit	1:4000	Santa Cruz (sc-253)	21800306, 30136719, 31423585	**F 7A–D, SF 3A–D, SF 4A–D**
TH (tyrosine hydroxylase)	Sheep	1:1000	Millipore (AB1542)	29888787	**SF 1F,L,N, SF 3A–D, SF 4A–D**
TH	Chicken	1:1000	Aves (TYH)	21858819, 21858821, 28472858, 30592042	N/A
VAChT (Vesicular acetylcholine transporter)	Goat	1:1000	Millipore Sigma (ABN100)	30926750	**F 4B1,2, SF 3A–D, SF 4A–D**
ChAT (Choline acetyltransferase)	Goat	1:1000	Millipore Sigma (AB144P)	26470751, 22237784, 16917846, 17111374, 22173709, 21618225	N/A
**SECONDARY**					
α-Guinea pig 488	Donkey	1:400	Jackson ImmunoResearch (706-545-148)		
α-Rabbit 594	Donkey	1:400	Jackson ImmunoResearch (711-585-152)		
α-Rabbit 488	Donkey	1:400	Jackson ImmunoResearch (711-545-152)		
α-Sheep Cy5	Donkey	1:400	Jackson ImmunoResearch (713-175-147)		
α-Sheep 488	Donkey	1:400	Abcam (ab150177)		
α-Chicken AMCA	Donkey	1:400	Jackson ImmunoResearch (703-155-155)		
α-Goat 594	Donkey	1:400	Jackson ImmunoResearch (705-585-147)		
α-Goat Cy5	Donkey	1:400	Jackson ImmunoResearch (705-175-147)		

**F, Figure; SF, Supplementary Figure.**

### Fluorescent *in situ* Hybridization Combined With Immunohistochemistry

To label GalR1 on GlyT2 neurons in the brainstem, multiplex FISH (RNAscope, ACDBio, Hayward, CA, United States) was performed (*n* = 5). Briefly, animals were deeply anaesthetized with sodium pentobarbital (70 mg/kg, ip), brains were isolated following transcardial perfusion with 0.1M PBS and embedded in OCT. Coronal sections (14 μm thick) were cut on a cryostat, mounted on superfrost plus slides (Thermo Fisher Scientific, Waltham, MA, United States) and stored at −80°C. Sections were fixed in 4% PFA/0.1M phosphate buffer, dehydrated and subjected to the RNAscope multiplex fluorescent assay according to the manufacturer’s instructions, in combination with fluorescence IHC. The riboprobes used were GalR1 (448821-C1) and GlyT2 (409741-C3). We also employed GalR2 (448831-C2) riboprobe, however, were unable to detect a signal anywhere in the brain using this probe. Positive and negative controls were incorporated into each procedure to verify RNA quality and specific staining. Slides were coverslipped with ProLong Gold Antifade Mountant with or without DAPI (Life technologies).

### Cell Counts and Analysis

A 1 in 6 series of 14 μm coronal sections and a 1 in 3 series of 30 μm coronal sections through the brain was examined for FISH and non-radioactive chromogenic ISH respectively. Immunostaining was examined under brightfield and epifluorescence microscopy using an Olympus BX51 equipped with a motor driven microscope stage, a digital camera (2000R-F-CLR) and a mercury powered light burner (Olympus U-LH100HG 100w). Neuronal profiles were plotted with using StereoInvestigator software version 9 (Microbrightfield, United States). Sections were aligned with reference to Bregma level according to a mouse stereotaxic brain atlas (Paxinos and Franklin, 2004). Only neurons with DAPI-stained nuclei were considered for counting and mapped. Furthermore, to decrease bias in the experimental results, the person quantifying experimental outcomes was blinded to the experimental groups. For quantitative analysis, the total cell counts were obtained following Abercrombie correction (Abercrombie, 1946): for 30 μm thick sections, an average nuclear width of 7.2 ± 0.2 μm and average section thickness of 29.7 ± 1 μm was measured from 30 cells and 10 sections respectively in 10 animals; for 14 μm thick sections, an average nuclear width of 7.9 ± 0.3 μm and average section thickness of 14 ± 1 μm was measured from 30 cells and 10 sections respectively in 5 animals. All values are given with ± SEM values. For semi-quantitative analysis, 5 sections per animal were screened from 5 animals per group. Regions were graded for expression density; - (not expressed), + (scattered sparsely), ++ (expressed by 1/3 of the neurons in the area), +++ (expressed by > 1/3 of the neurons in the area). Representative images were first exported into Fiji (RRID:SCR_002285) as TIFF files for brightness/contrast adjustment to increase the clarity and to reflect true rendering. Images were not otherwise altered. TIFF images were then imported into CorelDraw Graphics Suite X7 (64-bit) or Adobe Illustrator CC (2019) for final presentation.

## Results

### The Effect of Hypercapnia on Neuropeptide Expression in the Rostral RTN (rRTN)

**FIGURE 2:**
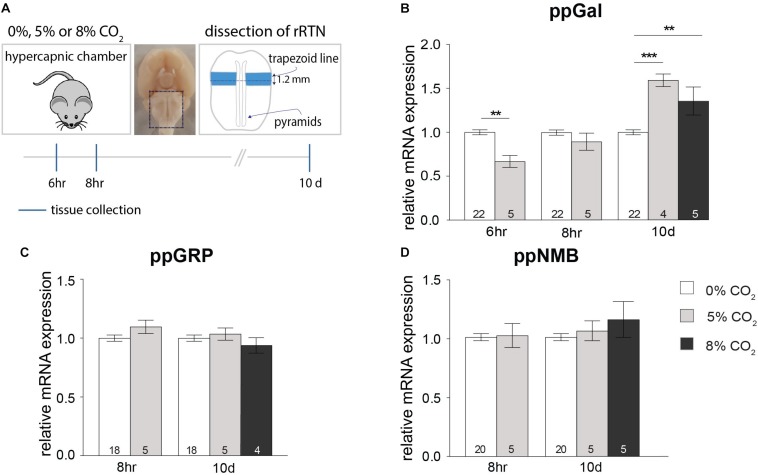
Short-term hypercapnia downregulates and long-term hypercapnia upregulates ppGal but not ppGRP or ppNMB mRNA in rostral RTN (rRTN) measured by qPCR. **(A)** Strategy for hypercapnia exposure, tissue dissection and timeline for short-term and long-term hypercapnia paradigms. **(B–D)** ppGal, ppGRP and ppNMB mRNA expression levels following short-term (6 or 8 h) or long-term (10 days) elevated inspired CO_2_ (5% or 8%). The qPCR results were normalized to the expression levels of two house-keeping genes: HPRT and RPLPO. ^∗∗^*p* < 0.01, ^∗∗∗^*p* < 0.001 (One-Way ANOVA, Multiple comparisons: Dunnet).

As a result of short-term (6 h), exposure to 5% CO_2_, ppGal expression significantly decreased in the rRTN (33%, *p* < 0.01) (Figure 2B). This decrease was no longer evident at the 8 h timepoint. In contrast, 10 days exposure to either 5 or 8% CO_2_ increased ppGal expression by 60% (*p* < 0.001) and 40% (*p* < 0.01) respectively (Figure 2B). There was no change to ppGRP nor ppNMB expression during either short-term or long-term hypercapnia (Figures 2C,D). Neuropeptide expression did not change in the lateral cerebellum.

### The Effect of Hypercapnia on Neuropeptide Expression in the Caudal RTN (cRTN)

**FIGURE 3:**
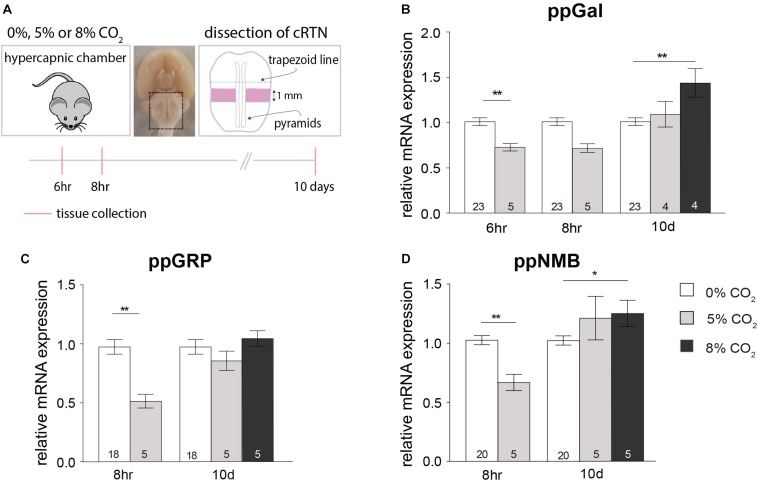
Short-term hypercapnia downregulates and long-term hypercapnia upregulates ppGal, ppGRP or ppNMB mRNA in caudal RTN (cRTN) measured by qPCR. **(A)** Strategy for hypercapnia exposure, tissue dissection and timeline for short-term hypercapnia and long-term hypercapnia paradigms. **(B–D)** ppGal, ppGRP and ppNMB mRNA expression levels following short-term (6 or 8 h) or long-term (10 days) elevated inspired CO_2_ (5% or 8%). The qPCR results were normalized to the expression levels of two house-keeping genes: HPRT and RPLPO. ^∗^*p* < 0.05, ^∗∗^*p* < 0.01 (One-Way ANOVA, Multiple comparisons: Dunnet).

Following a 6 h exposure to 5% CO_2_, there was a significant decrease in the expression of ppGal in the cRTN (27%, *p* < 0.01) (Figure 3B). After 8 h, the expression of ppGRP decreased by 49% (*p* < 0.01) and ppNMB by 33% (*p* < 0.01) (Figures 3C,D) with no change in ppGal expression. On the other hand, after long-term exposure to hypercapnia (8% CO_2_, 10 days), there was an increase in the expression of ppGal (40%, *p* < 0.01) and ppNMB (30%, *p* < 0.05) but not ppGRP.

### ppGal Distribution in the Mouse Brainstem

In the dorsal brainstem, ppGal+ neurons were abundant in the non-catecholaminergic caudal NTS (cNTS); ppGal+ neurons lay immediately ventral to the TH+ A2 neurons, within the lateral cNTS. They were also abundant in vAChT+ neurons in the dorsal motor nucleus of the vagus (X), but absent in hypoglossal nucleus (XII) (Supplementary Figure S3). There was no ppGal mRNA labeling observed in the rostral NTS (rNTS, rostral to area postrema). In the ventral brainstem, ppGal mRNA labeling was present in the RTN, inferior olive (IO) and paragiganticellular reticular nucleus. There was no ppGal mRNA labeling observed in the mouse VRC, A1/C1 populations (Supplementary Figure S4) or caudal raphe nuclei. The quantitative profile for the distribution of the ppGal in the RTN is illustrated in Supplementary Figures S2A,B. The total number of ppGal+ RTN neurons counted between bregma levels −6.78 to −5.70 mm was 398 ± 19 neurons (*n* = 30) per animal which corresponded to 50.2% of total RTN neurons (Phox2b+ /TH-/ChAT-) (Supplementary Figure S2B). Laterally, there was a moderate amount of ppGal+ neurons in the trigeminal nucleus (Sp5). In the rostral medulla, ppGal+ neurons were abundant in the locus coeruleus (LC) some of which were TH+. There was also a ppGal+/TH- population in proximity to A5 area, immediately lateral to the rostral pole of the facial nucleus (VII). In the context of respiratory populations in pons; parabrachial nucleus (PbN) and caudal Kölliker fuse (cKF) neurons did not contain ppGal mRNA. More rostrally in the pons, there was moderate amount of ppGal labeling in the area corresponding to rostral Kölliker fuse (rKF) and TH positive A4 population. Summary for semi-quantitative analysis of ppGal mRNA distribution in the brainstem is provided in Table 3.

**TABLE 3:** Distribution of ppGal and GalR1 mRNA in the mouse brainstem.

**Brainstem region**	**GalR1**	**ppGal**
Sp5	++	++
cNTS	+++	+++
rNTS	+++	−
XII	++	−
X	+	+++
IO	−	++
VRC	+	−
A1/C1	+	−
RTN	+	+++
na	+	−
Reticular nucleus	+	++
Caudal raphe nuclei	++	−
Lateral to A5	n/a	++
LC	+	+++
PbN	+++	−
cKF	+	−
rKF	+	+
A4	+	++

**Adult male C57BL6/J mice (n = 5) were examined using DIG-incorporated riboprobes complementary to mouse ppGal and GalR1 mRNA by ISH. All anatomical areas listed are according to the atlas of Franklin and Paxinos (Paxinos and Franklin, 2004). −, *not expressed; +, scattered sparsely; ++, expressed by 1/3 of the neurons in the area; +++, expressed by > 1/3 of the neurons in the area; n/a, not available.***

### GalR1 Distribution in the Mouse Brainstem

Both DIG-incorporated ISH and multiplex FISH approaches revealed high levels of GalR1 mRNA (GalR1+) in the PbN, cNTS and rNTS (Supplementary Figure S1 and Table 3). GalR1+ neurons in the NTS were Phox2b- and TH- (Supplementary Figures S1K,L). Low to moderate GalR1 mRNA labeling was detected in the Sp5, XII, VRC, A1/C1, RTN, nucleus ambiguus (na), reticular nucleus, raphe nuclei, and LC. The GalR1+ neurons of the RTN were also Phox2b+ (Supplementary Figures S1H,I). Upon quantitation, GalR1 mRNA labeling in the VRC shows two peaks: at Bregma levels of approximately 7.38 and rostral to −6.6 which correspond to the preBötzinger complex (preBötC) and Bötzinger complex (BötC) respectively (Figure 5D and Supplementary Figure S1O). An average of 242 ± 23 (*n* = 3) TH+ neurons were positive for GalR1 mRNA in the C1 area (Bregma levels −7.47 to −6.66) which corresponds to an average of 34% of all TH+ neurons in the C1 (Supplementary Figure S1P). Summary for semi-quantitative analysis for the distribution of GalR1 mRNA labeling and representative microscopic images are provided in Table 3 and Supplementary Figure S1 respectively.

### GalR1 Distribution in the Mouse Ventral Respiratory Column (VRC)

**FIGURE 4:**
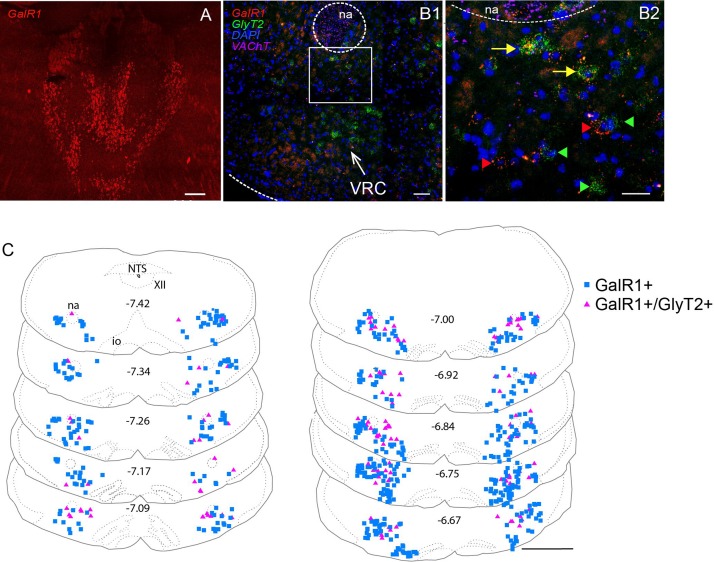
Distribution of GalR1 mRNA expressing neurons in the VLM. **(A)** The GalR1 FISH riboprobe demonstrated labeling of specific thalamic nuclei as described previously [Supplementary Figures S1A (adapted from Kerr et al., 2015), 1B]. **(B1)** GalR1 and GlyT2 mRNA labeling was observed ventral to nucleus ambiguus (na), in a region corresponding to the VRC. **(B2)** Inset in B1 enlarged, showing GalR1+ (red arrowhead), GlyT2+ (green arrowhead) and GalR1+/GlyT2+ double labeled (yellow arrow) neurons. **(C)** Representative mouse brainstem coronal sections, showing GalR1 and GlyT2 mRNA labeling from Bregma level −7.42 to −6.67. Scale bars are 200 μm **(A)**, 50 μm **(B1)**, 25 μm **(B2)** and 1 mm **(C)**.

**FIGURE 5:**
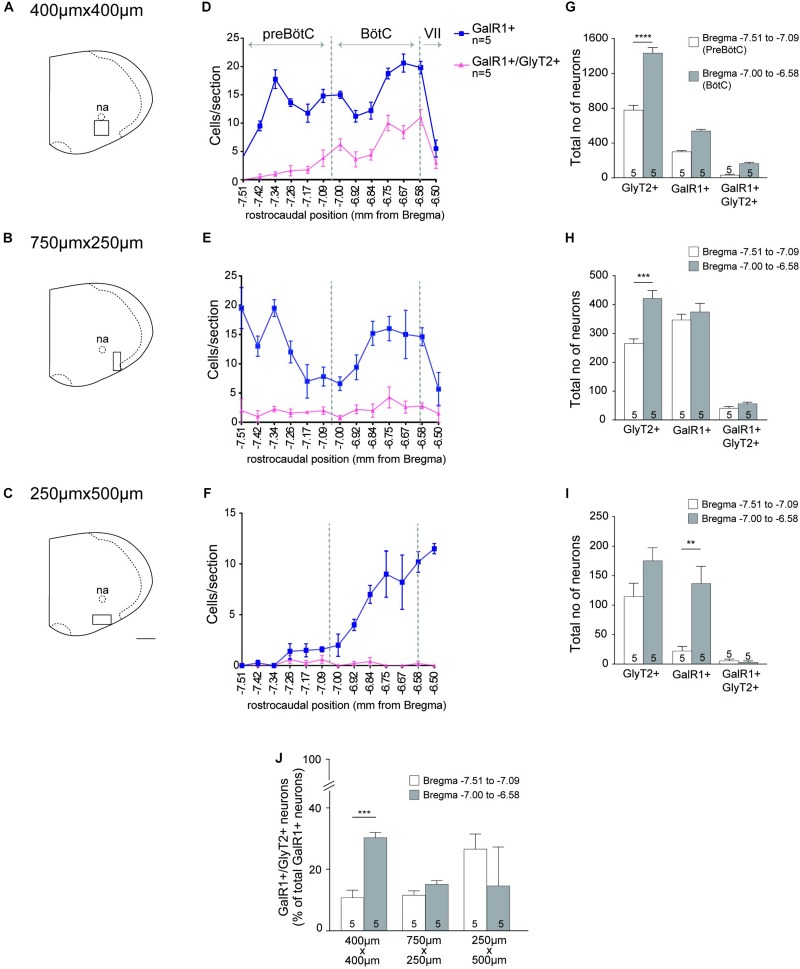
GalR1 mRNA is expressed in the glycinergic subset of VRC neurons. **(A–C)** Representative coronal hemisection of mouse brainstem showing three distinct areas analyzed (VRC immediately ventral to nucleus ambiguus (na); 750 × 250 μm region immediately lateral to the VRC; 500 μm × 250 μm region immediately ventral to the VRC). **(D–F**) Rostrocaudal distribution of GalR1 and GlyT2 mRNA containing neurons counted within the regions depicted in **(A-C)** respectively. **(G–I)** Total number of neurons per region of interest per mouse. PreBötC was defined to correspond to Bregma level −7.51 to 7.09 and BötC was defined to correspond to Bregma level −7.00 to −6.58. **(J)** Percentage of GalR1 neurons that are glycinergic in the region depicted in **(A–C)**. ^∗∗∗∗^*p* < 0.0001, ^∗∗∗^*p* < 0.001, ^∗∗^*p* < 0.01, ^∗^*p* < 0.05 (Two-Way ANOVA, Multiple comparisons: Tukey). Scale bar is 500 μm.

Inhibitory neurons in the VRC were identified by using a second riboprobe targeting GlyT2. Co-labeling of GalR1 with GlyT2 mRNA was apparent in the VRC (Figure 4B). Quantitative analysis for neurons containing both GalR1 and GlyT2 mRNA was performed in the ventral brainstem between Bregma levels −7.51 and −6.50. Based on previous studies (Abdala et al., 2015; Ausborn et al., 2018), preBötC and BötC correspond to Bregma levels −7.51 to 7.09 and −7.0 to −6.58 respectively. GalR1 distribution was not restricted to the VRC (Figure 4C). For this reason, cell counts were made from ventrolateral medulla (VLM) subdivisions (Figures 5A–C). The VRC was defined as a 400 μm^2^ region immediately ventral to the nucleus ambiguus (Pagliardini et al., 2003; Stornetta et al., 2003; Bouvier et al., 2010; Le et al., 2016; Figure 5A) and there was an average of 840 ± 23 (*n* = 5) GalR1+ neurons between Bregma levels −7.51 and −6.50 out of which 198 ± 15 were glycinergic (23%). In the VRC, BötC contained more GalR1+ neurons 539 ± 20 compared to preBötC 301 ± 12 (*n* = 5) (Figure 5G). The percentage of GalR1+ neurons expressing GlyT2 was also higher in BötC (30%) compared to preBötC (11%) (Figure 5J).

Lateral to the VRC (750 μm × 250 μm) (Figure 5B), the distribution of GalR1+ neurons had a similar pattern to the VRC (Figure 5E). In total, there were 721 ± 35 (*n* = 5) GalR1+ neurons between Bregma levels −7.51 and −6.50 (Figure 5H). The percentage of GalR1+ neurons expressing GlyT2 was also similar in both preBötC and BötC (12 and 15% respectively) (Figure 5J).

In the region ventral to the VRC (250 μm × 500 μm) (Figure 5C), the distribution pattern of GalR1 was different to the other two regions (Figure 5F). There was a significantly greater number of GalR1+ neurons in the BötC (137 ± 29 neurons) compared to preBötC (22 ± 8 neurons) (*p* < 0.005) (*n* = 5) (Figure 5I). Very few GalR1+ neurons were positive for GlyT2 in preBötC or BötC (Figure 5J). This population of GalR1 neurons most likely corresponds to the bulbospinal presympathetic population of rostral ventrolateral medulla.

### Chemoreflex Activation of Brainstem Neuronal Populations After Long-Term Hypercapnia

A semi-quantitative analysis was performed to identify regions that are differentially affected by acute chemoreceptor stimulation following room air or long-term hypercapnia. c-Fos immunoreactivity in the caudal raphe nuclei, the reticular nuclei (corresponding to lateral and ventral to the VRC), PbN and KF and did not change with acute hypercapnia chemoreflex challenge with or without long-term hypercapnia (Table 4). ppGal+ neurons in the Sp5, IO, reticular nucleus, rKF and A4 were not activated by any conditions (Table 4).

**TABLE 4:** c-Fos expression evoked by acute hypercapnia following room air or long-term hypercapnia conditions.

		**c-Fos expression**			
		**RA^∗^**	**RA + AH^∗^**	**LH^∗^**	**LH + AH^∗^**
**Brainstem region**		**(0% CO_2_)**	**(0% CO_2_ + AH)**	**(8% CO_2_)**	**(8% CO_2_ + AH)**
ppGal+	Sp5	−	−	−	−
	cNTS	−	+++	−	−
	IO	−	−	−	−
	RTN	+	+++	+++	+++
	Reticular nucleus	−	−	−	−
	LC	−	+	−	−
	rKF	−	−	−	−
	A4	−	−	−	−
ppGal−	rNTS	−	−	−	−
	A1/C1	−	++	−	−
	Caudal raphe nuclei	++	++	++	++
	PbN	++	++	++	++
	KF	++	++	++	++

*Adult male C57BL6/J mice (n = 5) were examined using c-Fos by IHC for activation and DIG-incorporated riboprobes complementary to mouse ppGal mRNA by ISH. All anatomical areas listed are according to the atlas of Franklin and Paxinos (Paxinos and Franklin, 2004). −, not expressed; +, scattered sparsely; ++, expressed by 1/3 of the neurons in the ROI; +++, expressed by > 1/3 of the neurons in the ROI. ^∗^RA, Room air; AH, acute hypercapnic chemoreflex challenge; LH, long-term hypercapnia.*

As described previously, discrete neuronal populations were activated by acute chemoreflex challenge (10% CO_2_-1 h). These included cNTS neurons, LC neurons, RTN neurons and A1/C1 neurons (*p* < 0.001). After 10 days of continuous 8% CO_2_ (long-term hypercapnia), we observed that many fewer cNTS, LC and A1/C1 neurons were recruited in response to acute hypercapnia chemoreflex challenge (Table 4 and Supplementary Figures S3, S4). The decreased activation of cNTS, LC, and C1 neurons suggests a blunting of the chemoresponsiveness of these regions after long-term hypercapnia.

### RTN Neurons Retain Chemoresponsiveness Following Long-Term Hypercapnia

The rostrocaudal distribution of RTN neurons (Phox2b+ /TH-/ChAT-) in the parafacial region is shown in Supplementary Figure S2A. In total, we counted 793 ± 41 neurons (*n* = 25) per mouse (Supplementary Figure S2B).

**FIGURE 6:**
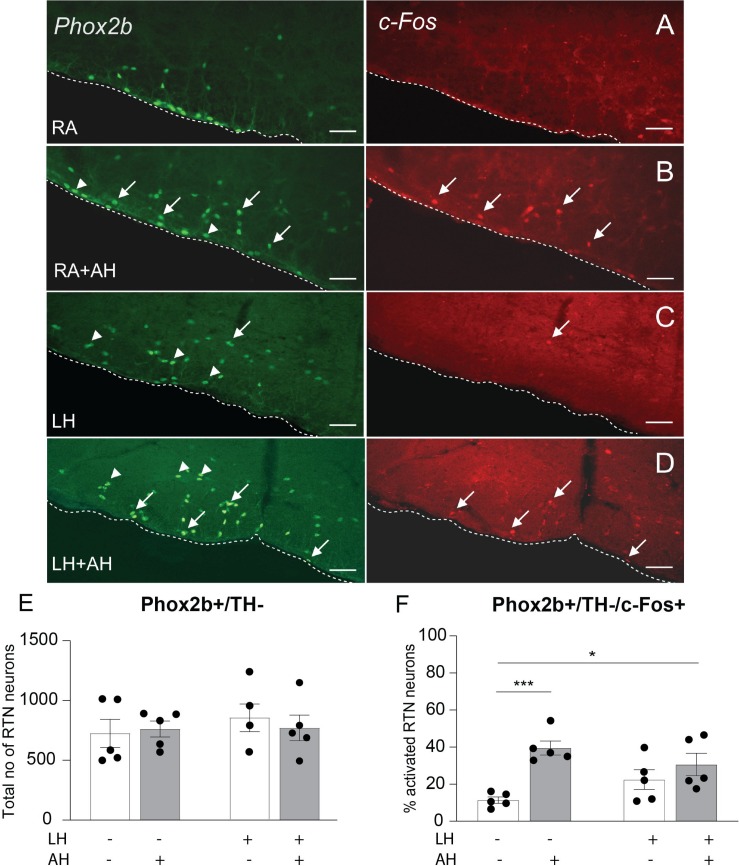
RTN neurons retain their responsiveness to acute hypercapnia chemoreflex challenge following long-term hypercapnia. Representative image of a coronal brainstem section from the RTN region for each experimental group immunostained for Phox2b (green) and c-Fos (red), **(A)** room air (RA) control, **(B)** room air + acute hypercapnia chemoreflex challenge (RA + AH), **(C)** long-term hypercapnia (LH), **(D)** long-term hypercapnia + acute hypercapnia chemoreflex challenge (LH + AH). Arrowheads point to non-activated RTN neurons; arrows point to activated RTN neurons. Scalebars are 25 μm. **(E)** The total number of RTN neurons (Phox2b+ /TH−) per mouse. **(F)** The percentage of activated RTN neurons following different experimental conditions. ^∗^*p* < 0.05, ^∗∗∗^*p* < 0.001 (Two-Way ANOVA, Multiple comparisons: Sidak).

Following control conditions of room air for 10 days, a baseline of 11.3% of RTN neurons displayed c-Fos immunoreactivity (84 ± 12, *n* = 5) (Figures 6A,F and Supplementary Figure S2C). By comparison, following the acute hypercapnia chemoreflex challenge, 39.5% of the RTN neurons were activated (275 ± 30 total cell counts) (*n* = 5) (Figures 6B,F and Supplementary Figure S2C), consistent with previous literature (Teppema et al., 1997; Kumar et al., 2015).

We observed a non-significant increase in c-Fos+ RTN neurons due to residual 8% CO_2_ from the 10 days exposure (11.3% vs. 22.4%, 84 ± 12 vs. 161 ± 35 neurons, *n* = 5) (Figures 6C,F and Supplementary Figure S2C). When the acute hypercapnia challenge was administered following the long-term hypercapnia exposure, the responsiveness of RTN neurons was significantly higher compared to room air control (30.7% vs. 11.3%, 242 ± 31 vs. 84 ± 12 total cell counts, *n* = 5) (Figures 6D,F and Supplementary Figure S2C). In summary, the RTN was the only population assessed that retained its chemoresponsiveness following long-term hypercapnia.

### ppGal+ RTN Neurons Retain Chemoresponsiveness Following Long-Term Hypercapnia

**FIGURE 7:**
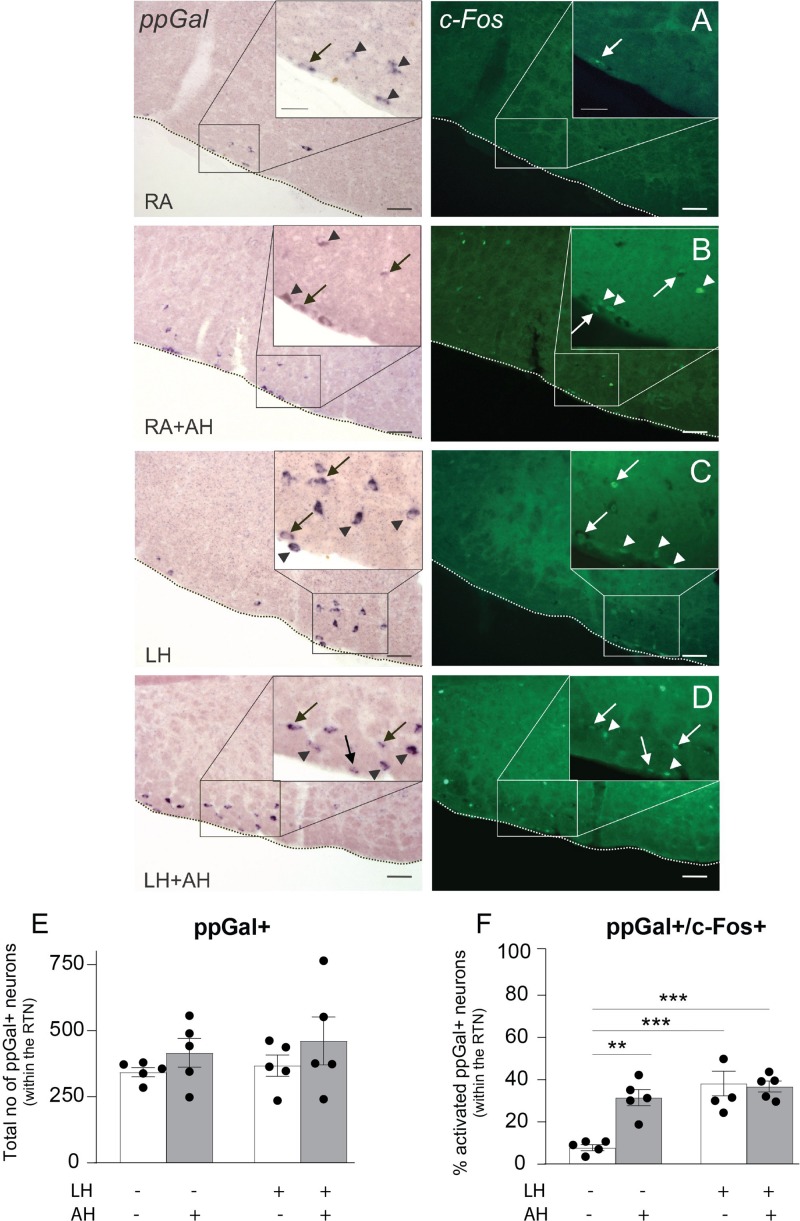
The ppGal+ neurons in the RTN do not change their responsiveness to acute hypercapnic chemoreflex challenge after a long-term exposure to hypercapnia. Representative images of the coronal brainstem sections from the RTN region showing the activation of the galaninergic RTN neurons following **(A)** room air (RA), **(B)** room air + acute hypercapnia chemoreflex challenge (RA + AH), **(C)** long-term hypercapnia (LH), **(D)** long-term hypercapnia + acute hypercapnia chemoreflex challenge (LH + AH). ppGal+ neurons are purple (alkaline phosphatase ISH labeled neurons) and c-Fos+ neurons are in green (IHC). The box in each panel is enlarged for a higher magnification representation of the area. Arrowheads point to single labeled neurons; arrows point to activated ppGal+ RTN neurons. Scale bars are 50 μm in low magnification images and 25 μm in high magnification images. **(E)** The total number of ppGal+ RTN neurons per mouse. **(F)** The percentage of activated ppGal+ neurons of total ppGal+ neurons within the RTN following different challenge conditions. ^∗∗^*p* < 0.01, ^∗∗∗^*p* < 0.001 (Two-Way ANOVA, Multiple comparisons: Sidak).

The ppGal+ subset of mouse RTN neurons displayed extensive c-Fos immunoreactivity in response to the acute hypercapnia challenge, consistent with previous descriptions in rat (Spirovski et al., 2012). Under control conditions (room air, 10 days), 26 ± 5 (*n* = 5) ppGal+ RTN neurons were activated (Figure 7A and Supplementary Figure S2D), representing 7.6% of the ppGal+ neurons (Figure 7F) which we infer to be 31% of the activated RTN neurons in the area. By comparison, following the acute hypercapnia challenge, 157 ± 33 ppGal+ RTN neurons were activated, representing 37.4% the ppGal+ neurons (*p* < 0.01) (*n* = 5) (Supplementary Figure S2D and Figures 7B,F) which we infer to be 57.1% of the activated RTN neurons in the area.

After long-term hypercapnia, there was no change in the number of ppGal+ RTN neurons activated by acute chemoreflex challenge (36.8% vs. 37.4% of ppGal+ RTN neurons, 167 ± 29 vs. 157 ± 33 total cell counts, *n* = 5) (Figures 7D,F and Supplementary Figure S2D). This was inferred to be 68.7% of the activated RTN neurons.

The qPCR results showed increased ppGal expression in both cRTN and rRTN following exposure to 8% CO_2_ for 10 days (Figures 2, 3). This could be explained by either an increase in the number of ppGal expressing neurons or increased expression of ppGal mRNA within each ppGal+ neuron. However, the number of ppGal+ RTN neurons did not change following long-term hypercapnia (Figure 7E). Furthermore, there was also no difference in the number of ppGal+ neurons when an analysis was conducted compartmentalizing RTN into rostral and caudal regions. Taken together, this suggests that whilst long-term hypercapnia does not induce more RTN neurons to express ppGal or alter the recruitment of ppGal+ neurons, it does increase the transcription of galanin mRNA.

## Discussion

The principal findings of this study are as follows: in the mouse, 50% of RTN neurons express galanin mRNA; gene expression for galanin was reduced by 30% following short-term hypercapnia and increased by 60% following long-term hypercapnia in both rostral and caudal RTN. In the mouse, galanin mRNA is distributed in brainstem regions important for cardiorespiratory regulation including cNTS and LC but in contrast to the rat, was absent in the VRC and A1 populations. Furthermore, the presence of GalR1 mRNA in the BötC and preBötC supports the existence of an RTN-VRC galaninergic circuit. Finally, our c-Fos studies showed that all brainstem galaninergic populations; RTN, NTS, LC, and some non-galaninergic populations such as A1 neurons were recruited by the acute hypercapnia chemoreflex challenge (indicated by increased c-Fos expression) which was consistent with previous literature (Spirovski et al., 2012). The CO_2_ responsiveness of all populations recruited (including galaninergic subpopulations) was blunted after long-term hypercapnia, however, RTN neurons retained their CO_2_ responsiveness. Taken together, there was both increased ppGal expression and sustained chemoresponsiveness of ppGal+ RTN neurons following long-term hypercapnia.

### Neuroplasticity Underlying Adaptation to Long-Term Hypercapnia

While it is well established that acute hypercapnia causes an increase in the ventilatory drive by peripherally and centrally mediated chemoreflex systems (Forster and Smith, 2010; Smith et al., 2010), the ventilation pattern is altered during long-term hypercapnia. The most common trend during long-term hypercapnia is a biphasic response consisting of an initial increase in ventilatory drive followed by a reduced response over the long term (Schaefer, 1963; Schaefer et al., 1963; Clark et al., 1971; Guillerm and Radziszewski, 1979; Jennings and Davidson, 1984; Burgraff et al., 2018, 2019). This time-dependent physiologic change suggests an adaptation or acclimatization to long-term hypercapnia. Peripheral homeostatic mechanisms including metabolic compensation by the kidneys are partly responsible for maintaining body pH by increasing HCO_3_^+^ levels (Schaefer, 1963; Schaefer et al., 1963; Johnson, 2017; Burgraff et al., 2018). Recently central mechanisms contributing to adaptation to long-term hypercapnia, including central/neural resetting, have been purported (Burgraff et al., 2019; Chuang et al., 2019). These studies suggest that changes in synaptic expression or changes in the phosphorylation of glutamate receptors contribute to this neuroplasticity. Since glutamate is the primary transmitter responsible for CO_2_ stimulated breathing (Bochorishvili et al., 2012; Holloway et al., 2015), glutamate receptors were good candidates for contributing to chronic hypercapnia-related neuroplasticity. Indeed, there are time dependent changes of glutamate receptor expression within cVLM, rRTN, rNTS, medullary raphe, rVRC and XII during chronic hypercapnia (Miller et al., 2014; Burgraff et al., 2019). These previous studies demonstrate that time and site-specific neuroplasticity occurs during long-term hypercapnia.

The data presented herein focuses on the RTN and proposes other neuromodulatory candidates that may contribute to neuroplasticity during long-term hypercapnia; neuropeptides galanin, NMB and GRP. Our qPCR results support the theory of a biphasic response to long-term hypercapnia; we found a decrease in neuropeptide mRNA expression in the first 6–8 h exposure, followed by an increase after 10 days. The decreased neuropeptide expression in the first phase aligns temporally with the initial hyperventilation and the increased neuropeptide mRNA expression in the second phase ultimately may reflect the neuroplastic change that underlies the decreased ventilation following long-term exposure to hypercapnia.

### Neuropeptide mRNA Expression in the RTN Is Altered in Response to Long-Term Hypercapnia

Our ppGal *in situ* hybridization data revealed ppGal mRNA expression in mouse brainstem is restricted to the RTN, IO, medial reticular nucleus, NTS and LC. In contrast to the rat, ppGal mRNA is absent in the VRC and A1/C1 populations of the mouse. Since the dissections separated ventral brainstem from dorsal brainstem and the midline region was excluded, “RTN” dissections were free from IO, medial reticular nucleus, NTS and LC ppGal mRNA. Therefore, we can confirm specificity of ppGal from the RTN in our dissections.

Previous studies showed that galanin levels do not change in whole brainstem homogenate after 3 h exposure to hypercapnia (Wang W. et al., 2013), however, here we have demonstrated 30% reduction in galanin gene expression following precise excision of the RTN. Since, galanin exerts a powerful inhibitory effect on the phrenic motor output by depressing the activity of neurons in the BötC and preBötC (Abbott et al., 2009a), the downregulation of ppGal mRNA we observed after 6 h would be expected to cause a consequent increase in phrenic motor output, facilitating hyperventilation.

Long-term hypercapnia did not change ppGRP mRNA expression, whereas ppGal and ppNMB mRNA levels were upregulated in the cRTN, and ppGal mRNA in the rRTN. Sustained increased activity of glutamatergic transmission was observed in the RTN and VRC following 30 days exposure to hypercapnia (Burgraff et al., 2019). It was shown that during the first hour of 6% CO_2_ exposure, there is an increase in minute ventilation by 355% of control (room air) levels, which decreases to 235% and was maintained over 30 days in goats (Burgraff et al., 2018). Upregulation of galanin mRNA expression in the RTN may contribute to the decrease in ventilation whereas increased NMB mRNA expression, an excitatory neuropeptide, may act as a counterbalancing mechanism to contribute to the sustained increased steady state minute ventilation after long-term hypercapnia compared to room air. In order to further support this hypothesis and establish a causal relationship, galanin co-release with glutamate and NMB can be investigated using electrophysiological and pharmacological approaches.

Neuropeptides are inducible and are known to have long-lasting effects since they act on G-protein coupled receptors (Salio et al., 2006; Nassel, 2009). Furthermore, they require progressively larger frequencies of stimulation for vesicular release (Fulop et al., 2005) and are released at synapses in response to higher frequencies of discharge than fast neurotransmitters (Balkowiec and Katz, 2000; Sauerstein et al., 2000). The increase in ppGal mRNA expression following exposure to long-term hypercapnia (5 and 8% CO_2_) may reflect increased neurotransmission.

### mRNA for Galanin Receptor 1 (GalR1) Is Distributed in the Mouse VRC

Since microinjection of galanin into the VRC results in an inhibitory effect on ventilation (Abbott et al., 2009a), we hypothesized that there would be GalR1 and 3 present in the VRC, because they are Gi/Go coupled and mediate inhibitory actions of galanin (Ogren et al., 2010). The present study demonstrates the presence of >840 GalR1+ neurons in the BötC and preBötC area.

A subpopulation (23%) of these GalR1+ neurons are glycinergic which may implicate a disinhibitory effect of galanin neurotransmission. However, photo- or pharmacological inhibition of the VRC is not obligatory for cessation of preBötC rhythmogenesis (Feldman and Smith, 1989; Sherman et al., 2015; Del Negro et al., 2018). Nevertheless, the inhibitory effect of galanin (Abbott et al., 2009a) following microinjection into the VRC might be through (1) indirect innervation of the phrenic motor nucleus by glycinergic VRC neurons (via XII, NTS, RTN, LC, PbN, KF, periaqueductal gray, colliculi nuclei, hypothalamus, thalamus, zona incerta, lateral preoptic area) (Yang and Feldman, 2018), or (2) GalR effects on other subtypes of VRC neurons since the phenotype of the remaining 77% of GalR1 neurons in VRC is unknown. The VRC also contains excitatory neurons (Del Negro et al., 2018). Therefore, there exists a complex circuitry between RTN galaninergic and target VRC neurons with the possibility of multiple combinations of galanin receptors and downstream excitatory or inhibitory VRC neurons which requires further elucidation.

### The Responsiveness of ppGal+ RTN Neurons to Acute Hypercapnia Challenge Is Not Altered Following Long-Term Hypercapnia

This study is also the first to look at the effect of long-term hypercapnia on the activation and responsiveness of galaninergic and non-galaninergic respiratory nuclei. There are multiple studies that looked at the alterations in c-Fos expression within central respiratory populations following acute (1–2 h) exposure to hypercapnia (5–15% CO_2_) or hypoxia (8–10% O_2_) (Teppema et al., 1997; Takakura et al., 2006; Spirovski et al., 2012; Wakai et al., 2015). Here we wanted to see if there are alterations in the responsiveness to acute hypercapnia chemoreflex challenge following long-term hypercapnia.

All the RTN neurons are vesicular glutamate transporter 2+ (Stornetta et al., 2009) and glutamate is the neurotransmitter used by the RTN during CO_2_-stimulated breathing (Holloway et al., 2015). Acute hypercapnia chemoreflex challenge activated ∼40% of ppGal+ RTN neurons which we inferred represents ∼60% of activated RTN neurons, in accordance with the previous literature (Stornetta et al., 2009). Our gene expression data shows a decrease in ppGal mRNA expression in the first 6 h of exposure to 5% CO_2_. A reduction in ppGal neurotransmission within the RTN may potentiate glutamatergic transmission, possibly contributing to the hyperventilatory response of the first phase of long-term hypercapnia.

The activation state of RTN neurons including the ppGal+ subset was sustained by the continuous 10 days exposure to 8% CO_2_. One limitation is that c-Fos is not an appropriate marker for long-term neuroplasticity because its protein expression peaks within 2 h of a stimulus challenge; it is a marker for transient neuronal activation and does not reflect long-term synaptic activity (Muller et al., 1984; Nestler et al., 2001). In future, ΔFosB, another member of the Fos family of transcription factors (Nestler et al., 2001), can be used to detect sustained changes in gene expression that persist long after exposure. ΔFosB accumulates during repeated activating stimuli thus whether RTN neurons are continuously responsive to the hypercapnia exposure over 10 days or not can be confirmed by the accumulation of the transcription factor ΔFosB.

### Unlike RTN, the Responsiveness of NTS, LC, and A1/C1 Neurons to Acute Hypercapnia Challenge Is Blunted Following Long-Term Hypercapnia

Our data demonstrating recruitment of cNTS neurons during acute chemoreflex challenge is in concert with the previous experiments conducted in rats where there was 10% increase in activation of ppGal+ NTS neurons (Spirovski et al., 2012). This might be due to either second order activation or intrinsic acid sensing nature of the NTS neurons. Focal acidification by CO_2_ dialysis with 25% CO_2_ increased ventilation *in vivo* (Nattie and Li, 2002) and increased the firing rate of Phox2B+ NTS neurons *in vitro* (Fu et al., 2017). Lesions of Phox2b containing NTS neurons by injections of neurotoxin substance P-saporin reduced minute ventilation and tidal volume during exposures to 4–8% CO_2_ (Fu et al., 2017). This transient activation of cNTS in response to acute hypercapnia chemoreflex challenge was lost following long-term hypercapnia.

The responsiveness to acute hypercapnia challenge of LC neurons was consistent with previous studies (Ritucci et al., 2005; Biancardi et al., 2008; Spirovski et al., 2012). This responsiveness is likely to be due to the intrinsic chemosensitive properties of the LC neurons. Indeed, local dialysis with acetazolamide increased ventilation (Biancardi et al., 2008), whereas focal deletion of these neurons decreased hypercapnic ventilatory response in unanesthetized rats (Biancardi et al., 2008). LC neurons are exclusively TH+ (Pickel et al., 1975); most ppGal+ neurons in the LC were TH+ as described previously (Dobbins and Feldman, 1994). Although not specifically characterized, these neurons may project to the VRC. Transsynaptic retrograde virus injections into the phrenic nucleus show third order LC afferents passing through the VRC (Holets et al., 1988). Similar to our findings with the NTS, LC neurons were responsive to acute hypercapnia chemoreflex challenge, and this responsiveness was blunted following long-term hypercapnia.

Similarly, the responsiveness of A1/C1 neurons to acute hypercapnia challenge following room air was consistent with the results reported by Spirovski et al. (2012) and Haxhiu et al. (1996). In rats, 52% of the ppGal+ neurons of the A1 population are activated by 2 h exposure to 10% CO_2_ even following carotid denervation (Haxhiu et al., 1996). Although A1 neurons do not express ppGal in mice, they remain responsive to acute hypercapnia challenge. Our data demonstrates for the first time that like NTS and LC, the responsiveness of A1/C1 neurons to chemoreflex challenge is blunted following long-term hypercapnia.

The blunting of respiratory related populations in response to the acute chemoreflex suggests an adaptation of the chemoreflex circuitry under chronic hypercapnic conditions. Our working hypothesis is that this type of adaptation would be advantageous for people with chronic respiratory diseases like COPD, OHS, and OSA who normally live under chronic hypercapnic conditions. The RTN was the only brain region assessed that remained recruited following long-term hypercapnia; this sustained chemoresponsiveness attests to its crucial role in central regulation of breathing. The conservation of ppGal+ RTN neurons that retained chemoresponsiveness following long-term hypercapnia suggests that the ppGal+ subpopulation is likely to be modulated during the adaptation process.

## Conclusion and Significance

Hypercapnia is a respiratory stressor that occurs in many disease (e.g., COPD, OHS, OSA, etc.) or non-disease conditions (e.g., miners, submarines, scuba divers, astronauts, hibernating animals, deep sea creatures, etc.). While the physiologic mechanism that underlies the central respiratory chemoreflex response to long term hypoxia is well established, the mechanisms underlying central neuroplastic changes that occur during long-term hypercapnia are yet to be clarified. This is the first study assigning a role to the neuropeptides galanin, NMB and GRP in the RTN to adaptive changes during long-term hypercapnia. The major findings are as follows: (1) Neuropeptide gene expression decreased after short-term hypercapnia and increased after long-term hypercapnia with galanin showing the most prominent changes. This supports the concept of a biphasic neuroplastic contribution during adaptation to long-term hypercapnia. (2) For the first time, GalR1 mRNA was shown to be present in the BötC and preBötC supporting the existence of an RTN-VRC galaninergic circuitry. (3) Finally, c-Fos studies showed that after exposure to long-term hypercapnia, the responsiveness of the NTS, LC and A1 populations to acute hypercapnia chemoreflex challenge was blunted. In contrast RTN neurons including galaninergic subpopulation retained their responsiveness to acute hypercapnia. Co-release of galanin from RTN neurons may (1) counterbalance glutamatergic inputs to the respiratory centers in order to downscale energetically wasteful hyperventilation in the first phase of chronic respiratory stress (hypercapnia) (2) have a role in the second phase of adaptation to long-term hypercapnia, contributing to the observed decrease in ventilation through the inhibitory effects of galanin.

## Data Availability Statement

All datasets generated for this study are included in the article/Supplementary Material.

## Ethics Statement

Experiments were performed on adult male wild type (C57BL6/J) mice, reviewed and approved by the University of New South Wales Animal Care and Ethics Committee and conducted in accordance with the Australian Code for the Care and Use of Animals for Scientific Purposes (National Health and Medical Research Council of Australia).

## Author Contributions

NK conceptualized the research and provided critical revisions. NK and AD designed the research, with the guidance of NK, AD, and ZY conducted all experiments and acquired data. NK, AD, ZY, and PC analyzed, interpreted results of experiments, and approved final version of the manuscript. AD prepared the figures and drafted the manuscript.

## Conflict of Interest

The authors declare that the research was conducted in the absence of any commercial or financial relationships that could be construed as a potential conflict of interest.

## Abbreviations

4V: 4th ventricle
AH: acute hypercapnic chemoreflex challenge
AP: area postrema
BötC: Bötzinger complex
CO_2_: carbon dioxide
A1/C1: catecholaminergic populations of A1 and C1 area
c-,r-: caudal and rostral
cc: central canal
CL, CM, MHb, MDM, PC, PV: central lateral, central medial, medial mediodorsal, medial habenula, paracentral, paraventricular thalamic nuclei
ChAT: choline acetyltransferase
COPD: Chronic Obstructive Pulmonary Disease
comm-,m-,l-, commissural, medial: lateral NTS
DG: dentate gyrus
X: dorsal motor nucleus of vagus
VII: facial nucleus
FISH: fluorescent *in situ* hybridization
GalR1: galanin receptor 1
GRP: gastrin releasing peptide
GlyT2: glycine transporter 2
HKG: house-keeping gene
XII: hypoglossal nucleus
HPRT: hypoxanthine-guanine phosphoribosyltransferase
IHC: immunohistochemistry
ISH: *in situ* hybridization
KF: Kölliker Fuse
LC: locus coeruleus
LH: long-term hypercapnia
NMB: neuromedin b
N_2_: Nitrogen
na: nucleus ambiguous
NTS: nucleus of the solitary tract
OHS: obesity hypoventilation syndrome
OSA: obstructive sleep apnea
O_2_: oxygen
Phox2b: paired − like homeobox 2b
PbN: parabrachial nucleus
PFA: paraformaldehyde
preBötC: preBötzinger complex
pp-: prepro-
py: pyramids
qPCR: quantitative polymerase chain reaction
RPLPO: ribosomal protein lateral stalk subunit P0
RA: room air
SH: short-term hypercapnia
scp: superior cerebral peduncle
Sp5: trigeminal nucleus
TH: tyrosine hydroxylase
VRC: ventral respiratory column
VLM: ventrolateral medulla
VAChT: vesicular acetylcholine transporter
