# Supplementary material for: Adaptation of Respiratory-Related Brain Regions to Long-Term Hypercapnia: Focus on Neuropeptides in the RTN
Source: Front Neurosci. 2019 Dec 13;13:1343. doi: 10.3389/fnins.2019.01343 (PMC6923677; doi:10.3389/fnins.2019.01343)
Supplement: Supplementary file 1 [file Data_Sheet_1.pdf]

Supplementary Material

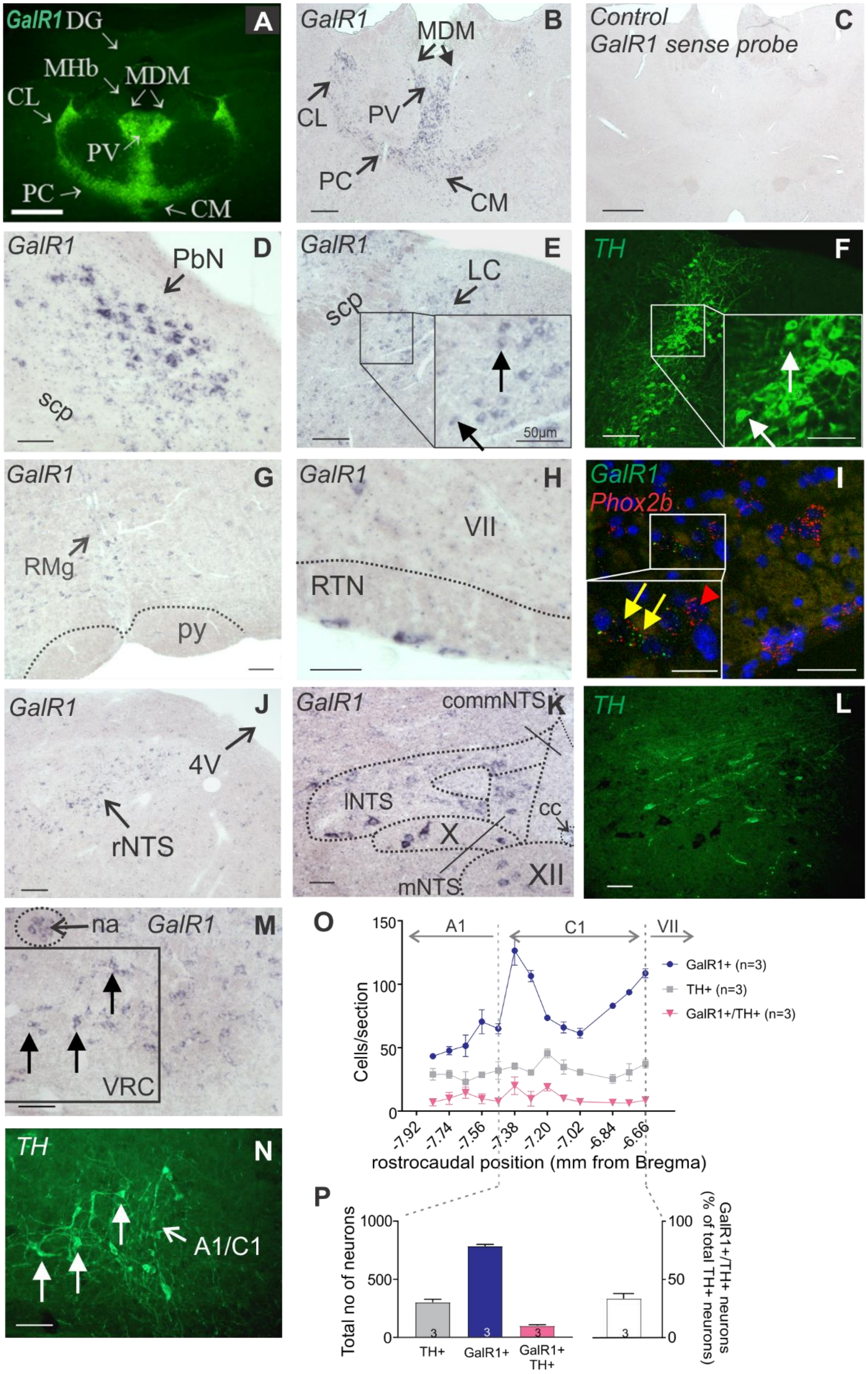

GalR1 DG MHb MDM CL PV PC CM A
GalR1 MDM CL PV PC CM B
Control GalR1 sense probe C
GalR1 PbN scp D
GalR1 LC scp 50µm E
TH F
GalR1 RMg py G
GalR1 VII RTN H
GalR1 Phox2b I
GalR1 4V rNTS J
GalR1 commNTS K lNTS X cc mNTS XII
TH L
na GalR1 M VRC
TH N A1/C1
O
A1 C1 VII
Cells/section
GalR1+ (n=3)
TH+ (n=3)
GalR1+/TH+ (n=3)
-7.92 -7.74 -7.56 -7.38 -7.20 -7.02 -6.84 -6.66
rostrocaudal position (mm from Bregma)
P
Total no of neurons
TH+ GalR1+ GalR1+ TH+
GalR1+/TH+ neurons (% of total TH+ neurons)

Supplementary Figure 1 Distribution of GalR1 mRNA in the mouse brainstem. **(A)** A figure adapted from (Kerr et al., 2015) showing GalR1+ immunofluorescence in the thalamus of a GalR1 knock-in mouse [paraventricular- (PV), medial mediodorsal- (MDM), central medial- (CM), paracentral- (PC), central lateral- (CL) and intermediodorsal (IMD)-thalamic nuclei, but not in dentate gyrus (DG) or medial habenula (MHb)]; **(B)** GalR1 mRNA labelling the same thalamic regions as shown in panel A by Kerr et al. (2015) with high specificity; **(C)** There is no GalR1 mRNA labelling detected with the control sense riboprobe that was generated from the same sequence. There was abundant GalR1 mRNA labelling in specific areas of the brainstem including **(D)** parabrachial nucleus (PbN), **(E)** Locus coeruleus (LC), where some GalR1+ neurons (black arrows) were TH+ (white arrows) as shown in **(F)**; **(G)** raphe magnus (RMg), **(H)** RTN which is supported by fluorescent ISH (I) that depicted Phox2b only neurons (red arrowheads) and GalR1 and Phox2b double labelled neurons (yellow arrows); **(J)** rostal NTS (rNTS); **(K)** caudal NTS [commissural (comm), lateral (l), medial (m) NTS], dorsal motor nucleus of vagus (X), hypoglossal nucleus (XII). **(L)** In the NTS, none of the GalR1 containing neurons were catecholaminergic (TH+). **(M-N)** In the VLM, ventral respiratory column (VRC), nucleus ambiguus (na), A1 and C1 catecholaminergic tyrosine hydroxylase (TH) populations were GalR1+, TH neurons were immunolabelled with green fluorescence as shown by arrows. **(O)** Rostrocaudal distribution of GalR1 mRNA and TH protein containing neurons. Ordinate indicates mean +/- SE number of neurons per section (n=5). Sections were 90 µm apart from each other. Abscissa indicates Bregma level. **(P)** The number of GalR1 mRNA and TH protein containing neurons/section per hemisection was added to obtain total number of cells in C1 area and % of TH+ neurons that are GalR1+ (Bregma levels -7.47 to -6.66). The scale bars are 200 µm for the panels A and B, 500 µm for the panel C, 100 µm for the panels E-G and J, 50 µm for the panels D, H-I, K-N and the insets in E-F and 25 µm for the inset in I. Other abbreviations: superior cerebral peduncle (scp), pyramids (py), fourth ventricle (4V), central canal (cc).

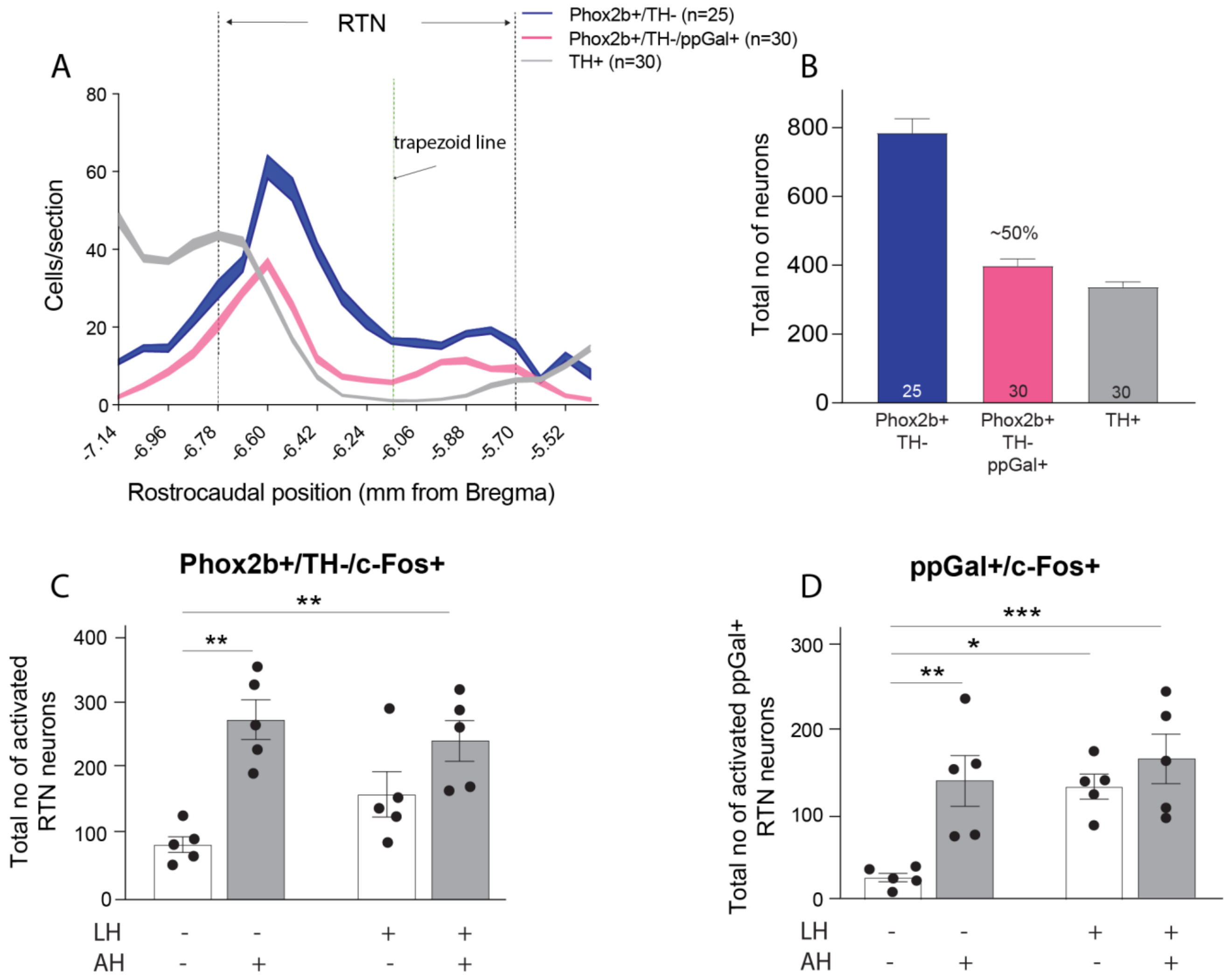

Supplementary Figure 2 The ppGal+ subpopulation of neurons in the RTN do not change their responsiveness to acute hypercapnia chemoreflex challenge after a long-term exposure to hypercapnia. **(A)** Average total numbers of RTN neurons (Phox2b+/TH-), the proportion that are ppGal+ (Phox2b+/TH-/ppGal+) and TH+ neurons in the ventrolateral medulla (VLM) per 30 µm thick coronal section. **(B)** The total number of RTN neurons (Phox2b+/TH-, blue), ppGal+ RTN neurons (pink) and TH+ neurons (grey) per mouse (as indicated by gray, dashed vertical lines in A). 50% of RTN neurons were ppGal+ **(C-D)** The total number of activated (c-Fos+) RTN neurons **(C)** and ppGal+ RTN neurons (D) per mouse following different experimental conditions: room air (RA), room air+acute hypercapnia chemoreflex challenge (RA+AH), long-term hypercapnia (LH), long-term hypercapnia+acute hypercapnia chemoreflex challenge (LH+AH). Abscissa for A indicates rostrocaudal location of tissue sections; distance from Bregma (Paxinos and Franklin, 2004). *p<0.05, **p<0.01, ***p<0.001, ****p<0.0001 (Two-Way ANOVA, Multiple comparisons: Sidak).

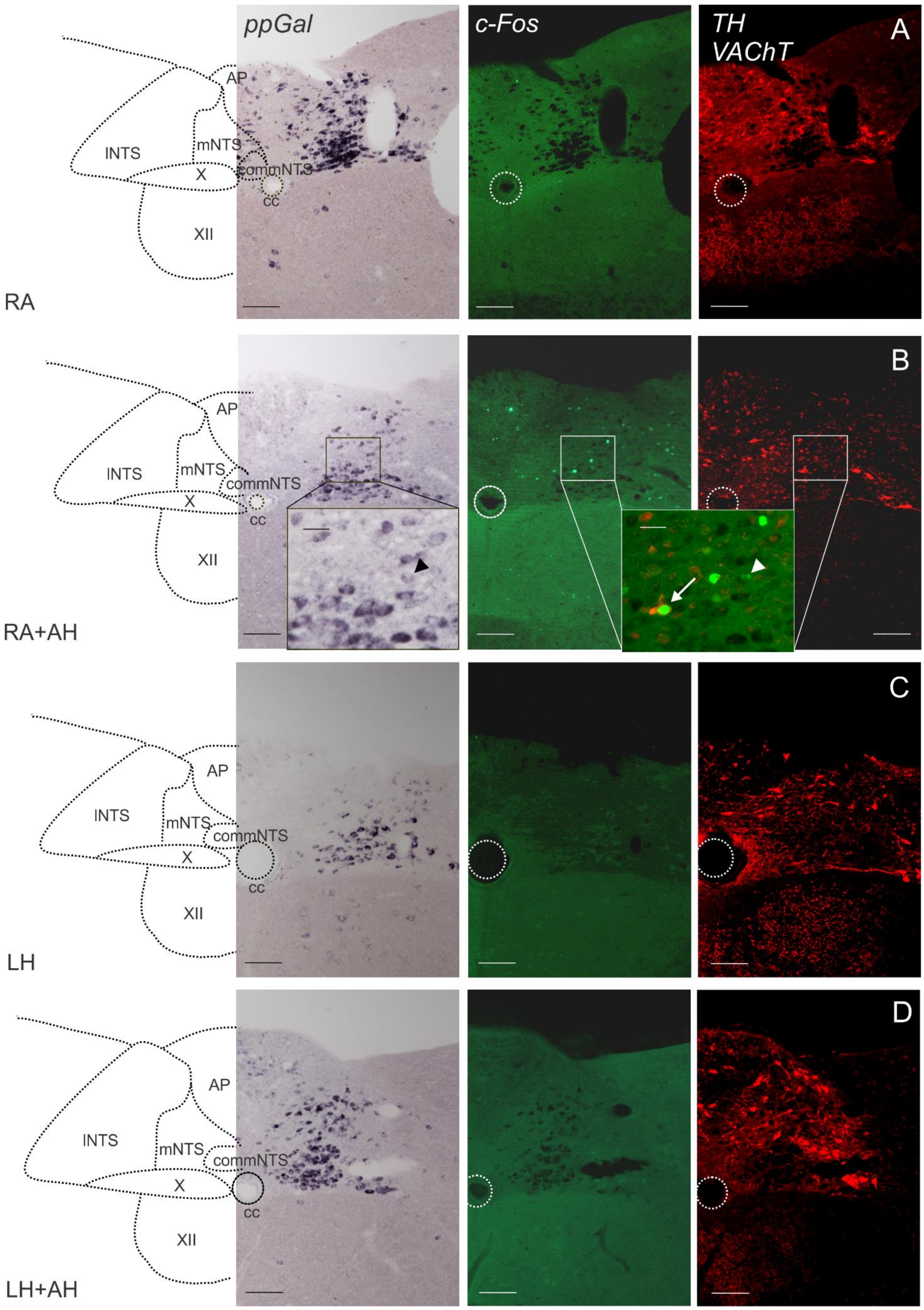
ppGal
c-Fos
TH
VAChT
A
B
C
D
AP
INTS
mNTS
commNTS
X
cc
XII
RA
RA+AH
LH
LH+AH

Supplementary Figure 3 Representative images of coronal brainstem sections from the cNTS region showing activation of galaninergic neurons following (**A**) room air (RA), (**B**) room air+acute hypercapnia chemoreflex challenge (RA+AH), (**C**) long-term hypercapnia (LH), (**D**) long-term hypercapnia+acute hypercapnia chemoreflex challenge (LH+AH). Preprogalanin (ppGal+) neurons are in purple (alkaline phosphatase ISH labelled), c-Fos+ neurons are in green (IHC), tyrosine hydroxylase (TH) and vesicular acetylcholine transporter (VAChT) neurons are in red (IHC). The inset is enlarged for a higher magnification representation of the area. The arrowhead points to an activated ppGal+ NTS neuron, the arrow points to an activated TH+ NTS neuron, Scale bars are 100 µm for the low magnification images and 25 µm for the inset. Abbreviations: commissural (comm), medial (m), lateral (l), nucleus of the solitary tract (NTS), central canal (cc), area postrema (AP), dorsal motor nucleus of vagus (X), hypoglossal nucleus (XII).

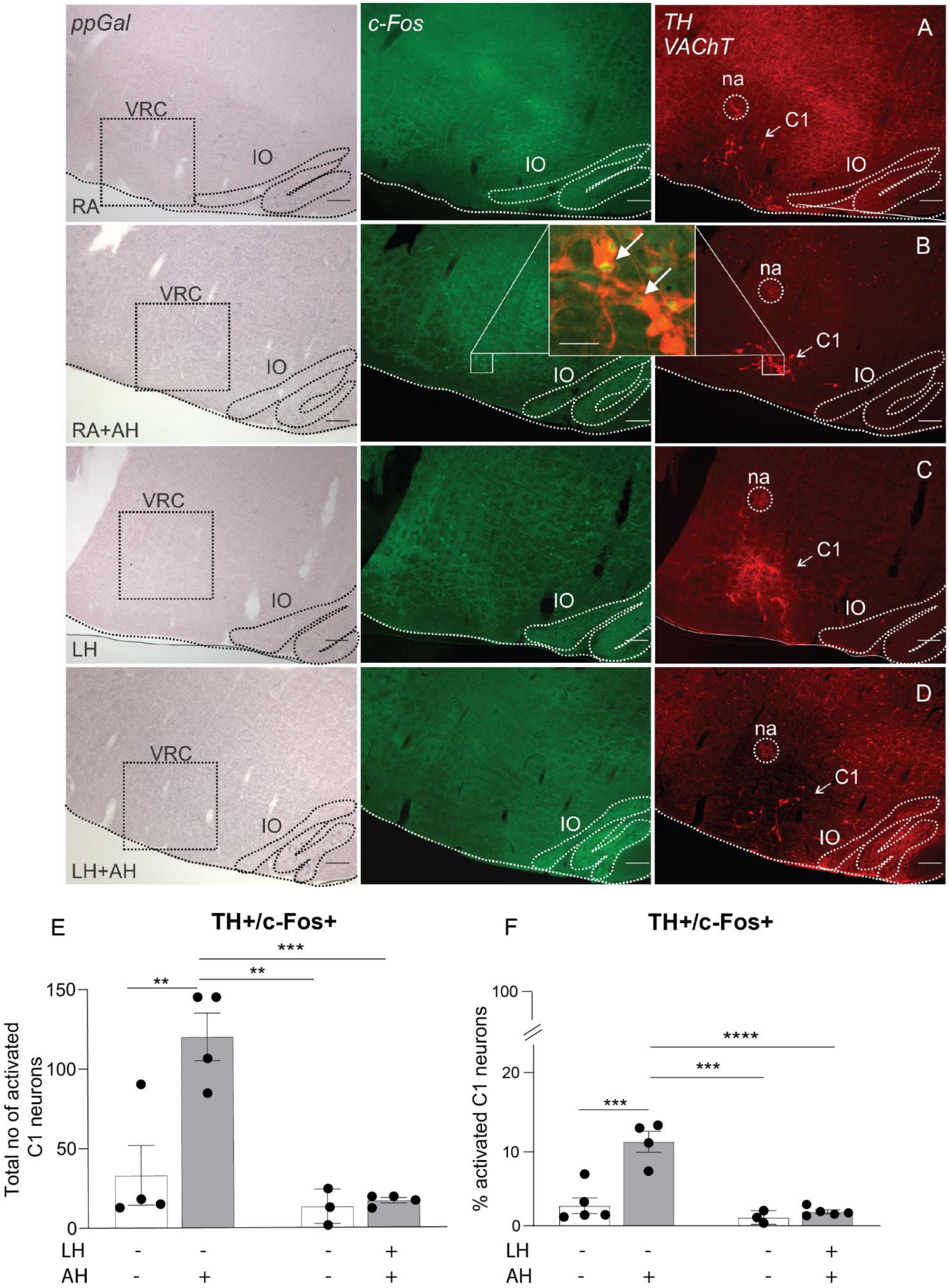

ppGal
c-Fos
TH
VAChT
A
B
C
D
VRC
IO
RA
RA+AH
LH
LH+AH
na
C1
E
TH+/c-Fos+
Total no of activated C1 neurons
150
100
50
0
**
***
LH
AH
F
TH+/c-Fos+
% activated C1 neurons
100
20
10
0
****

Supplementary Figure 4 Representative images of the coronal brainstem sections from the A1/C1 region showing the activation of the catecholaminergic (TH) neurons following **(A)** room air (RA), **(B)** room air+acute hypercapnia chemoreflex challenge (RA+AH), **(C)** long-term hypercapnia (LH), **(D)** long-term hypercapnia+acute hypercapnia chemoreflex challenge (LH+AH). Preprogalanin (ppGal+) neurons are in purple (alkaline phosphatase ISH labelled neurons), c-Fos+ neurons are in green (IHC) and TH and vesicular acetylcholine transporter (VAChT) neurons are in red (IHC). The dotted box is where the ventral respiratory column (VRC) located. The solid box inside the panel is enlarged for a higher magnification representation of the area. The arrows point to activated TH+ C1 neurons. The circled area is the nucleus ambiguus (na) labelled with VAChT in red (IHC). Scale bars are 100 µm for the low magnification images and 25 µm for the inset. **(E)** The total number of activated (c-Fos+) TH+ C1 neurons within the VLM (per mouse) following different experimental conditions. **(F)** The percentage of activated TH+ neurons of total TH+ C1 neurons following different experimental conditions. $^{**}p<0.01$, $^{***}p<0.001$, $^{****}p<0.0001$ (Two-Way ANOVA, Multiple comparisons: Sidak). Other abbreviations: inferior olive (IO).
